# Improving precision of glomerular filtration rate estimating model by ensemble learning

**DOI:** 10.1186/s12967-017-1337-y

**Published:** 2017-11-09

**Authors:** Xun Liu, Ningshan Li, Linsheng Lv, Yongmei Fu, Cailian Cheng, Caixia Wang, Yuqiu Ye, Shaomin Li, Tanqi Lou

**Affiliations:** 1 Division of Nephrology, Department of Internal Medicine, The Third Affiliated Hospital of Sun Yat-sen University, Guangzhou, 510630 China; 2 Division of Nephrology, The 3rd Affiliated Hospital of Sun Yat-sen University, Yuedong Hospital, Meizhou, 514700 China; 3 SJTU-YALE Joint Center for Biostatistics, Shanghai JiaoTong University, Shanghai, China; 4 Operating Room, The Third Affiliated Hospital of Sun Yat-sen University, Guangzhou, China; 5 Emergency Department, The Third Affiliated Hospital of Sun Yat-sen University, Guangzhou, China

**Keywords:** Chronic kidney disease, Glomerular filtration rate, Ensemble learning, Prediction, Precision

## Abstract

**Background:**

Accurate assessment of kidney function is clinically important, but estimates of glomerular filtration rate (GFR) by regression are imprecise.

**Methods:**

We hypothesized that ensemble learning could improve precision. A total of 1419 participants were enrolled, with 1002 in the development dataset and 417 in the external validation dataset. GFR was independently estimated from age, sex and serum creatinine using an artificial neural network (ANN), support vector machine (SVM), regression, and ensemble learning. GFR was measured by 99mTc-DTPA renal dynamic imaging calibrated with dual plasma sample 99mTc-DTPA GFR.

**Results:**

Mean measured GFRs were 70.0 ml/min/1.73 m^2^ in the developmental and 53.4 ml/min/1.73 m^2^ in the external validation cohorts. In the external validation cohort, precision was better in the ensemble model of the ANN, SVM and regression equation (IQR = 13.5 ml/min/1.73 m^2^) than in the new regression model (IQR = 14.0 ml/min/1.73 m^2^, P < 0.001). The precision of ensemble learning was the best of the three models, but the models had similar bias and accuracy. The median difference ranged from 2.3 to 3.7 ml/min/1.73 m^2^, 30% accuracy ranged from 73.1 to 76.0%, and P was > 0.05 for all comparisons of the new regression equation and the other new models.

**Conclusions:**

An ensemble learning model including three variables, the average ANN, SVM, and regression equation values, was more precise than the new regression model. A more complex ensemble learning strategy may further improve GFR estimates.

**Electronic supplementary material:**

The online version of this article (10.1186/s12967-017-1337-y) contains supplementary material, which is available to authorized users.

## Background

Determining glomerular filtration rate (GFR) is essential for screening early impairment of kidney function, evaluating progressive kidney deterioration and complications, adjusting the dosage of drugs, and managing the risks of chronic kidney disease (CKD) [1]. The gold standard GFR assays are inulin clearance and isotope imaging. The high cost of inulin limits its routine clinical use. Isotope imaging is moderately costly, but usage is limited by radiation exposure. Consequently, GFR is often estimated by regression analysis with sex, age and specific serum filtration markers (e.g., serum creatinine and cystatin C) as covariates. However, even the most accurate GFR estimation equations [2] are biased and imprecise [3] because they do not include physiological variables that only affect serum filtration markers other than GFR [4]. The performance of equations that estimate GFR may be improved either by adding additional covariates, or by applying more sophisticated or advanced method than traditional regression.

Ensemble learning is a simple, well-known machine learning method in which the results obtained with different mathematic models is combined to generate a better result. It is widely used in engineering applications because of its superior performance in estimation [5]. A previous study found that ensemble learning significantly outperformed single learning models for cataract detection and grading [6]. We hypothesized that performance of regression-based estimated GFR models can be improved by ensemble learning because it is more robust, allowing more complex relationship between GFR and covariates and can account for more complicated interactions among the covariates.

## Methods

### Data sources

The study enrolled consecutive participants in the Third Affiliated Hospital of Sun Yat-sen University, China. Some of the data evaluated in this study was previously evaluated and published [7]. Additional data obtained from participants with the same inclusion and exclusion criteria became available [7] and is shown in Additional file 1: Fig. S1. Of the 1815 participants screened, 1419 were included in the study and data collected from 1002 participants from January 2010 to June 2013 were evaluated in the development set. Data obtained from 417 participants from January 2005 to December 2009 were evaluated in the external validation set. The Institutional Review Board at the Third Affiliated Hospital of Sun Yat-sen University approved the study, and participants gave informed consent before inclusion.

### Laboratory methods

GFR was measured by 99mTc-DTPA renal dynamic imaging, and calibrated with a dual plasma sample 99mTc-DTPA GFR as previously described: dual plasma sample DTPA GFR (ml/min/1.73 m^2^) = (0.167 + 1.057) × DTPA by renal dynamic imaging − GFR [8]. Serum creatinine was by an enzymatic method with a Hitachi 7180 auto analyzer (Hitachi, Tokyo, Japan) and reagents from Roche Diagnostics (Mannheim, Germany). After the year 2010 serum creatinine was recalibrated to comply with the isotope dilution mass spectrometry reference method.

### Mathematical methods

The GFR estimation models were constructed using serum creatinine, age, and sex as covariates and eGFR as output. The three methods used were regression, artificial neural network (ANN) and support vector machine (SVM), and all generated mathematic models to describe the relationship between GFR and the covariates. ANN and SVM are widely used machine learning methods that can accommodate complex and nonlinear relationships that regression cannot. These three models were constructed using the development data set were used to estimate individual GFRs. The ensemble learning model was generated by averaging the output of the three single models. The construction of these models is described in detail in Table 1, Additional file 2: Item S1, Additional file 3: Item S2 and Additional file 4: Item S3. The performance of these four models was evaluated with the external validation data set.

**Table 1:** The regression equation used in this study

Gender	Serum creatinine (mg/dl)	Equation for estimating GFR
Female	≤ 1.2	$92 \times (SC \div 1.2)^{ - 0.534} \times 0.994^{Age}$
Female	> 1.2	$79 \times (SC \div 1.2)^{ - 0.516} \times 0.994^{Age}$
Male	≤ 1.0	$98 \times (SC \div 1.0)^{ - 0.450} \times 0.996^{Age}$
Male	> 1.0	$105 \times (SC \div 1.0)^{ - 0.640} \times 0.993^{Age}$

### Statistical analysis

The bias, precision and accuracy of the models were evaluated. Bias was the median difference of the measured GFR (mGFR) and eGFR; precision was the interquartile range (IQR) of the difference. Accuracy was the proportion of participants whose eGFR did not deviate more than 30% from the mGFR. The 95% confidence intervals (CIs) were calculated by the bootstrap method (2000 bootstraps) [9]. The regression model was used as the benchmark for comparison with the ANN, SVM, and ensemble models. Significance testing of the differences in performance was by the Wilcoxon signed rank test for medians, the bootstrap method for IQR, and the McNemar test for accuracy. Statistical significance level was P < 0.05. All statistical calculations were performed using R version 3.0.2 and MATLAB version 2011b.

## Results

### Study population

The baseline characteristics of the two datasets differed significantly (Table 2). The mean (± SD) mGFR was 70 ± 30 (range 10–174) ml/min/1.73 m^2^ in the development dataset compared with (53 ± 27 ml/min/1.73 m^2^, P < 0.001) in the external validation dataset. Mean age, body mass index and body weight were also lower in the developmental dataset, mean serum creatinine level and height were higher. There were fewer patients with diabetes and more men in the external validation data set.

**Table 2:** Patient characteristics in the development and validation datasets

Characteristic	Development (N = 1002)	External validation (N = 417)	P value^#^
Age (years)	55.7 ± 15.0	51.3 ± 16.0	< 0.001
Male proportion	570 (56.9)	262 (62.8)	0.044
Diabetes	500 (49.9)	97 (23.2)	< 0.001
Body mass index (kg/m^2^)	24.0 ± 3.7	22.9 ± 3.6	< 0.001
Weight (kg)	63.7 ± 12.3	61.5 ± 11.8	0.002
Height (cm)	162.5 ± 8.3	163.6 ± 7.6	0.020
Body-surface area (m^2^)	1.7 ± 0.2	1.7 ± 0.2	0.107
Serum creatinine (mg/dl)	1.7 ± 1.8	2.7 ± 2.5	< 0.001
Measured GFR			
Mean (ml/min/1.73 m^2^)	70.0 ± 29.6	53.4 ± 26.5	< 0.001
< 15 (ml/min/1.73 m^2^)	10 (1.0)	9 (2.2)	< 0.001
≥ 15 and < 30 (ml/min/1.73 m^2^)	99 (9.9)	94 (22.5)	
≥ 30 and < 60 (ml/min/1.73 m^2^)	275 (27.4)	149 (35.7)	
≥ 60 and < 90 (ml/min/1.73 m^2^)	345 (34.4)	123 (29.5)	
≥ 90 (ml/min/1.73 m^2^)	273 (27.2)	42 (10.1)	

Unless otherwise noted, data are reported as N (percentage); continuous variables are mean ± standard deviation

*GFR* glomerular filtration rate

^#^P values were derived from paired-sample t test

### Overall performance of GFR estimation models in the external validation data set

As shown in Table 3, the ensemble model was more precise (13.5 ml/min/1.73 m^2^) than the regression equation (14.0 ml/min/1.73 m^2^, P < 0.001). All models had similar bias and accuracy. Bias ranged from 2.3 to 3.7 ml/min/1.73 m^2^ and accuracy ranged from 73.1 to 76.0% (P > 0.05 for all comparisons between the regression equation and the other models).

**Table 3:** Bias, precision and accuracy of each model in the external validation data set

Variable	Measured GFR (ml/min/1.73 m^2^)			
	Overall	< 30	≥ 30 and < 60	≥ 60
Bias = median difference (95% CI)				
Regression model	2.3 (1.0–3.4)	4.4 (2.9–5.9)	3.1 (1.5–6.5)	−1.9 (−4.5 to 0.9)
ANN model	3.2 (2.2–5.4)	5.4 (3.1–7.4)	5.4 (2.4–7.6)	0.8 (−3.9 to 2.7)
SVM model	3.6 (2.6–4.9)	6.8 (4.9–9.0)^‡^	4.0 (2.2–6.4)	−0.2 (−2.3 to 2.6)
Ensemble model	3.4 (2.3–4.4)	5.6 (3.7–8.2)	4.0 (2.1–6.7)	−0.5 (−3.9 to 2.3)
Precision = IQR of the difference (95% CI)				
Regression model	14.0 (12.4–15.9)	9.2 (7.3–11.8)	13.5 (11.2–18.0)	19.6 (16.8 to 23.5)
ANN model	15.1 (13.6–17.0)^‡^	11.1 (9.1–14.8)^‡^	14.9 (13.1–17.7)^‡^	20.5 (17.9 to 25.1)^‡^
SVM model	14.2 (12.4–16.0)^‡^	9.5 (7.5–12.1)^‡^	12.9 (10.3–16.2)^‡^	18.5 (14.9 to 21.5)^‡^
Ensemble model	13.5 (11.8–14.9)^‡^	8.9 (7.0–11.0)^‡^	12.7 (10.4–16.0)^‡^	17.9 (15.44 to 21.9)^‡^
Accuracy = 30% accuracy (95% CI)				
Regression model	75.1 (70.7–79.4)	52.4 (42.7–61.2)	75.2 (67.8–81.9)	89.1 (83.6 to 93.3)
ANN model	73.4 (69.0–77.2)	54.4 (44.7–64.1)	70.5 (63.11–77.2)	87.9 (82.4 to 92.1)
SVM model	73.1 (68.8–77.2)	47.6 (37.9–57.3)	71.1 (63.11–77.9)	90.9 (86.1 to 94.5)
Ensemble model	75.5 (71.5–79.6)	52.4 (42.7–62.1)	73.8 (65.11–79.9)	91.5 (86.7 to 95.2)

*GFR* glomerular filtration rate, *ANN* artificial neural network, *SVM* support vector machine, *IQR* interquartile range, *CI* confidence interval

^‡^ *P* < 0.05 compared with regression model-GFR

### Subgroup analysis of performance of GFR estimation models in the external validation data set

The performance of the estimation models was also evaluated in subgroups of the external validation data set stratified by GFR (< 30, 30–60, and ≥ 60 ml/min/1.73 m^2^). The precision of the ensemble model was consistently better than that of the regression model (8.9 vs. 9.2 with GFR < 30, 12.7 vs. 13.5 with GFR 30–60, and 17.9 vs. 19.6 with GFR ≥ 60, all P < 0.001). Bias and accuracy were similar for all models across all GFR subgroups. There were no significant difference between the regression equation and the other models, except for the SVM model, in which bias was worse than the regression model in the GFR < 30 subgroup.

## Discussion

Regression, ANN, SVM, and ensemble learning models were developed to estimate GFR using sex, age, and serum creatinine as covariates. A single demonstration population was used to develop the models, and a single external validation data set was used to compare performance with that of the regression model. Precision was improved in the ensemble learning method, but bias and accuracy in the regression and the ensemble learning models were similar.

Ensemble learning has previously been used to estimate GFR. Inker et al. evaluated cystatin C as an additional covariate [2], finding that a model averaging creatinine and cystatin C values had similar bias (median difference) but higher precision (IQR of difference) and accuracy (the proportion of participants whose eGFR deviated from mGFR by more than 20 or 30%) than either of the two covariates used alone. In a later study, Inker et al. [10] used ensemble learning to evaluate β-trace protein (BTP) and β_2_-microglobulin (B2M) as covariates to estimate GFR. Their existing CKD-EPI creatinine–cystatin C equation, and a new equation including BTP and B2M were evaluated. The precision and accuracy of average of the two equations was similar to those of the individual equations. The ensemble learning method in those two studies used different combinations of covariates. In this study, ensemble learning employed different models all with the same covariates. There was no need to perform additional testing to measure additional covariates from each patient, which did not increase patient costs.

Why did the ensemble learning model perform better than the regression model? ANN and SVM models could have relatively low bias relative to variance [11, 12]. A good single model might be constructed from theoretical data, but the expected performance might not be realized with the datasets and parameter values used during the training process. A single model might, unfortunately have a high variance, but combining several models should decrease the overall variance, as the deviations of the single models would be in different directions [5]. Ensemble learning thus results in a high level of performance together with improved variance.

Ensemble learning performed better than the other models in GFR estimation even though simple averaging was used. Other, methods more complex than averaging such as weighted averaging or stacking have been used [5]. Planning for ensemble learning when designing single models will achieve increase diversity, leading increased performance improvement after model combination. It is to be expected that using more complicated ensemble learning strategies will further improve performance in GFR estimation.

The fact that the ensemble model achieved better precision but worse bias and accuracy than current regression model, demonstrated that it was difficult to develop a new model superior to regression model in all terms of bias, precision and accuracy, especially with only three covariates. Recently deep learning has made big success in medical informatics, but the constructed models are complicated with many covariates. That is, the advantages of complicated models are obvious in dealing with complex problem such as natural language processing, image and speech recognition. However, GFR estimation as a simple regression task limits the capacity of complicated models. It’s supposed that adding more relevant but different covariates may be more promising than improving method of model construction.

The study limitations include the evaluation of data representing a specific group of CKD patients in China. Also, although there were some differences in the characteristics of the patients in the development and the external validation cohorts, both were recruited at same institution. Consequently, external validation of the effectiveness of the ensemble learning method that was used is desirable.

## Conclusions

A novel ensemble learning model was developed to estimate GFR, and it achieved better precision than the widely used regression model. The advantage of ensemble learning method in estimation of GFR warrants further investigation.

## Additional files

**Additional file 1: Figure S1.** Trial profile.

**Additional file 2: Item S1.** ANN model calculator. Open the excel worksheet and change the covariate values to estimate GFR.

**Additional file 3: Item S2.** SVM model calculator. Open the excel worksheet and change the covariate values to estimate GFR.

**Additional file 4: Item S3.** Additional material.
