# Supplementary material for: Improving precision of glomerular filtration rate estimating model by ensemble learning
Source: J Transl Med. 2017 Nov 9;15:231. doi: 10.1186/s12967-017-1337-y (PMC5679185; doi:10.1186/s12967-017-1337-y)
Supplement: Supplementary file 1 — Additional file 1: Figure S1. Trial profile. [file 12967_2017_1337_MOESM1_ESM.doc]

**Screening data from 2005 to 2009**

**(N=515 participants)**

**Excluded: N=244**

**On dialysis (N=10)**

**Reversible kidney functcion (N=234)**

**Excluded: N=98**

**On dialysis (N=23)**

**Reversible kidney functcion (N=75)**

**Screening data from 2010 to 2013 (N=1246 participants)**

**Development data set**

**(N=1002)**

**External validation data set**

**(N=417)**

Figure S1: Trial profile
