# Supplementary material for: Improving precision of glomerular filtration rate estimating model by ensemble learning
Source: J Transl Med. 2017 Nov 9;15:231. doi: 10.1186/s12967-017-1337-y (PMC5679185; doi:10.1186/s12967-017-1337-y)
Supplement: Supplementary file 4 — Additional file 4: Item S3. Additional material. [file 12967_2017_1337_MOESM4_ESM.doc]

*Additional file 4: Item S3*

*Normalization of variables*

When developing artificial neural network (ANN) and support vector machine (SVM) models, variables needed to be normalized according to mean and standard deviation of corresponding variable from development data set. Normalization formula was:

$$z=\frac{x-\overline{x}}{s}$$

where z was value of one variable after normalization, x was the original value of that variable, $\overline{x}$ and s was mean and standard deviation of that variable calculated only from development data set, respectively. Continues variables (Serum creatinine, age and GFR) were normalized respectively, while normalization was unneeded for gender as it was a category variable.

*Development of the ANN Model*

Whole structure of ANN

A 3-variable feedforward neural network was constructed, with serum creatinine, age, and gender as the covariates and estimated GFR (eGFR) as the only output variable. Serum creatinine, age and GFR were normalized according to previous formula. Gender was encoding as a binary variable, that is male equaled 1 while female equaled 0. The number of neurons of input layer was the number of input variable which equaled 3, and the number of neurons of output layer was the number of output variable which equaled 1. Since GFR estimation could be model as a regression problem, 2 two-hidden-layer network was chosen other than one-hidden-layer network, as two-hidden-layer network could learn more complicate function relationship between covatiates and output variables [1, 2]. The number of neurons of each hidden layer was set the same. Each neuron of hidden layers took Sigmod function as activation function, while the only neuron of output layer took identity function. An additional regularization parameter was used to control overfitting.

Training ANN

ANN was trained based on development data set. A superior combination of numbers of neurons of hidden layer in the network and values of regularization parameter was achieved by “trial and error”. 10-fold cross-validation was applied during superior parameter selection, that is, the whole development data set was equally divided into 10 subsets, with each time 9 subsets for training ANN and the remaining 1 subset for internal validation. A superior combination of structure parameter was the one with least average mean square error on internal validation. Finally, a 3-6-6-1 network with a regularization parameter 0.01 was selected, and was trained based on whole development data set.

The ANN was constructed using MATLAB software (The MathWorks Inc). Each network was initialized randomly, and trained by the algorithm of gradient descent and error back propagation. The error during training was minimized using fmincg function, which is a script written by Carl Edward Rasmussen. An Excel file (Item S1) showed the detailed construction of the new ANN model.

*Development of the SVM Model*

The covariates, output variable and the variable normalization process of the SVM were the same as those in the ANN model. Gender was encoding as a vector other than a number to improve performance, that is male was encoding into (1, 0) while female was encoding into (0, 1). SVM was constructed in MATLAB using libsvm package [3]. As GFR estimation was represented as a regression problem, epsilon-SVR (support vector regression) model was used, which is an extension of SVM from classification to regression. Kernel function was specified as radial basis function (RBF), which was a natural wildly-used function with good performance in common situation. There were three important parameters that must to be decided. One was epsilon, the parameter in loss function of epsilon-SVR model, which was specified as default value (0.1). Grid search method was used to decide superior values of other two parameters, penalty parameter or regularization parameter C and kernel parameter γ. 10-fold cross-validation was also used in the optimization of parameters. Detailed description of cross validation was demonstrated in the ANN construction part, and SVM construction used the same strategy. As complete fine grid search was time-consuming, a coarse grid was used first, and the result was C = 4096 and γ = 0.0625. Then a fine grid search on the region around the values was conducted. Finally parameter C was specified as 8192 and γ was 0.0526. A final model was constructed with this parameter set based on the whole development data set. Also an Excel file (Item S2) was developed to show the detailed construction of the new SVM model.

*Development of the Regression Model*

The regression model (Table 2) was developed from the development data set of this study. The equation used age, gender, and serum creatinine as the covariates. Serum creatinine knots for men and women were selected by the form of smoothing function as for the CKD-EPI equation [4]. The coefficients for serum creatinine, age and gender were estimated by least squares.

*Ensemble of the three models*

Ensemble method of ANN, SVM and regression equation was a simple averaging strategy, that is, the output of ensemble model, eGFR, was an average value of outputs of ANN, SVM and regression equation. The calculation formula was as follows:

$$eGFR=\frac{O_{ANN}+O_{SVM}+O_{regression}}{3}$$

where eGFR was the output of ensemble model. O_ANN_, O_SVM_ and O_regression_ represented the output of ANN, SVM and regression equation, respectively.

Reference

1. Haykin S. Neural Networks and Learning Machines (3rd Edition). Pearson Education, Inc; 2008.

2. Mitchell Tom. Machine Learning. McGraw Hill; 1997.

3. Chang CC, Lin CJ. LIBSVM: A Library for Support Vector Machines. *Acm transactions on intelligent systems and technology* 2011; 2: 27:21-27:27

4. Levey AS, Stevens LA, Schmid CH *et al.* A new equation to estimate glomerular filtration rate. *Ann Intern Med* 2009; 150: 604-612
